# Supplementary material for: Content-rich biological network constructed by mining PubMed abstracts
Source: BMC Bioinformatics. 2004 Oct 8;5:147. doi: 10.1186/1471-2105-5-147 (PMC528731; doi:10.1186/1471-2105-5-147)
Supplement: Additional File 2 — The original results of the above study (non-essential files are deleted to keep the file size under the limit set by BMC bioinformatics). [file 1471-2105-5-147-S2.bz2 › chilibotAdditionalFile2/dip10/548ID8872471E10901/html/AR_GAPD.html]

 


 **AR** and **GAPD** 
  
Found 11 abstracts in PubMed, retrieved 10.  
 

 What does Google say? 
 PDF only 
| .edu only 

---

**Interactive relationship** (e.g. stimulation, inhibition, etc)

**Neutral relationship**- The androgen receptor  [ **AR** ]  interacts with GAPDH  [ **GAPD** ]  both in the yeast two hybrid system and in vitro.  Ref: 8872471 Hum Mol Genet, 1996
- **AR**  transcript ranged from 302 to 1440 copies per 10 5 GAPDH  [ **GAPD** ]  copies and from 257 to 3223 copies per 10 5 GAPDH  [ **GAPD** ]  copies in the epithelium and stroma, respectively.  Ref: 11911259 Anticancer Res< MedlineTA>Anticancer Res, 1996

**Non-interactive relationship** (e.g. studied together, co-existance, homology, etc.)

- The binding of both ataxin 1 and the androgen receptor  [ **AR** ]  to GAPDH  [ **GAPD** ]  does not vary with the length of the polyglutamine tract.  Ref: 8872471 Hum Mol Genet, 1996
- In contrast, ARG and HB EGF mRNAs showed a coordinate pattern of expression in 7 10 specimens that was distinct from all other growth factor or receptor genes examined and from mRNAs for prostate specific antigen, the androgen receptor  [ **AR** ]  and GAPDH  [ **GAPD** ] , a house keeping enzyme.  Ref: 10579352 Endocrinology, 1999
- To investigate if GAPDH  [ **GAPD** ]  might interact with other glutamine repeat containing proteins involved in neurodegenerative disorders, we tested its binding to the androgen receptor  [ **AR** ]  which is mutated in spinobulbar muscular atrophy.  Ref: 8872471 Hum Mol Genet, 1996
- We have analyzed the expression of androgen receptor  [ **AR** ]   **AR** , progesterone receptor PR, estrogen receptor ER, clusterin CLU, complement C3 C3, and GAPDH  [ **GAPD** ]  mRNA in the rat uterus following oral administration of ethinylestradiol EE, bisphenol A BPA, o, p DDT DDT, p tert octylphenol OCT and daidzein DAI.  Ref: 10822019 J Steroid Biochem Mol Biol, 2000
- Inter and intra assay variability of gene quantitation was reflected by mean values of the variance coefficients of Ct of 4.84 1.13% and 2.73 0.39% or 3.32 1.03% and 2.21 0.24% corresponding to percent variances of copy numbers of 83.07 12.72% and 34.45 9.03% or 47.40 8.59% and 23.83 3.16% for human beta3  **AR**  and GAPDH  [ **GAPD** ]  genes, respectively.  Ref: 11735259 J Mol Cell Cardiol, 2001
- Primers and probes were designed to amplify the three subtypes of human beta adrenoceptors beta1, beta2 and beta3  **AR** , as well as reference genes such as glyceraldehyde 3 phosphate dehydrogenase  [ **GAPD** ]  GAPDH  [ **GAPD** ] , Hypoxanthine guanine phosphoribosyltransferase HPRT, and the oncogene ABL by real time RT PCR.  Ref: 11735259 J Mol Cell Cardiol, 2001
- On the other hand, the gene expression of alpha1b  **AR**  subtype did not change over the different stages in relation to that of a housekeeping gene, glyceraldehyde 3 phosphate dehydrogenase  [ **GAPD** ] , and to total  **AR** .  Ref: 11369312 Arch Oral Biol, 2001
